# Measurement of multiple biomarkers in advanced stage heart failure patients treated with pulmonary artery catheter guided therapy

**DOI:** 10.1186/cc11440

**Published:** 2012-07-25

**Authors:** Jodi L Zilinski, Ravi V Shah, Hanna K Gaggin, Mary Lou Gantzer, Thomas J Wang, James L Januzzi

**Affiliations:** 1 Cardiology Division, Department of Medicine, Massachusetts General Hospital, 55 Fruit Street, Boston, MA 02114, USA; 2 Siemens Healthcare Diagnostics, 500 Gbc Drive, Newark, DE 19702, USA

## Abstract

**Introduction:**

This study was carried out to investigate the prognostic utility of biomarkers in advanced stage heart failure (HF) patients requiring ICU admission for pulmonary artery catheter (PAC) guided therapy.

**Methods:**

Thirty patients admitted to an ICU for PAC guided HF therapy were enrolled; concentrations of soluble ST2 (sST2), highly sensitive troponin I, an experimental ultrasensitive troponin I, amino-terminal pro-B type natriuretic peptide, cystatin C, and myeloperoxidase were measured over the first 48 hours. Outcomes included response of filling pressures and hemodynamics to tailored therapy and 90-day event-free survival (death, left ventricular assist device implantation, transplant).

**Results:**

Of the biomarkers evaluated, only sST2 concentrations were higher in those who failed to achieve goals for central venous pressure ((CVP), 225.3 versus 104.6 ng/mL; *P *= 0.003) and pulmonary capillary wedge pressure ((PCWP), 181.7 versus 88.2 ng/mL; *P *= 0.05). Only sST2 concentrations were associated with adverse events (186.7 versus 92.2 ng/mL; *P *= 0.01). In age-adjusted Cox proportional hazards analysis, an elevated sST2 during the first 48 hours following ICU admission independently predicted 90-day outcomes (Hazard Ratio = 5.53; *P *= 0.03) superior to the Simplified Acute Physiology Score for this application; in Kaplan-Meier analysis the risk associated with elevated sST2 concentrations was present early and sustained through the duration of follow-up (log rank *P *= 0.01).

**Conclusions:**

In patients undergoing HF therapy guided by invasive monitoring, sST2 concentrations were associated with impending failure to reduce filling pressures and predicted impending events. Elevated sST2 values early in the ICU course theoretically could assist therapeutic decision-making in advanced stage HF patients.

**Trial registration:**

ClinicalTrials.gov Identifier: NCT00595738

## Introduction

The rising incidence of heart failure (HF) accounts for a rapidly increasing rate of hospitalization and death for those afflicted, with a consequent effect on overall costs of health care; each year, approximately $24 billion is spent on HF hospitalizations [1]. A large percentage of these cost outlays are due to patients suffering from advanced stages of HF. Although advanced stage HF patients comprise only a small portion of the total affected population, they have a very high risk of in-hospital morbidity and mortality and require the most intensive resources [2,3]. In one study of patients with New York Heart Association (NYHA) Class IV HF, ICU costs alone accounted for a quarter of the total expenditures during the final six months of life [4]. In this context, earlier and more specific recognition of elevated risk could theoretically guide clinicians' selection of more advanced therapies tailored to the baseline risk of the patient, potentially streamlining their care.

One increasingly-used option for risk assessment in HF patients is biomarker testing. Ventricular dysfunction and elevated filling pressures in severe HF trigger a cascade of deleterious pathophysiologic responses including inflammation, tissue remodeling, cardiorenal syndrome and neurohormonal dysregulation [5], all of which are associated with adverse outcome. The natriuretic peptides (for example, B-type natriuretic peptide (BNP) and its amino terminal cleavage fragment [NT-proBNP]) are the most widely used diagnostic and prognostic biomarkers in HF [6-8]. Additionally, concentrations of troponin, the interleukin receptor family member soluble (s)ST2 [9-11], renal measures such as cystatin C (cys-C) [12], and inflammatory markers such as myeloperoxidase (MPO) [13,14] have all been linked to adverse risk in HF, presumably as they reflect these various deleterious processes in such patients. While the potential value of various biomarkers for prognosis has been explored in detail across a wide spectrum of patients with HF, their specific prognostic value in affected patients at more advanced stages of HF, in whom decisions for advanced HF interventions frequently hinge on prognosis, remains less well explored. In theory, the use of biomarkers might be useful to decide at an earlier stage, prior to failure of invasive means of treatment, such as pulmonary artery catheter (PAC) guided therapy, whether a patient may require more direct referral for left ventricular assist device (LVAD) implantation or transplantation.

Accordingly, we evaluated a wide range of biomarkers reflecting various pathophysiologies in HF. We wished to determine the individual or additive role of each biomarker in predicting response to PAC guided therapy, as well as their relationship to short-term outcome following ICU admission.

## Materials and methods

All patients provided written informed consent and the study protocol detailed below and as described in ClinicalTrials.gov NCT00595738 was approved by the Partners Human Research Committee institutional review board. The original goal of the study was to evaluate the relationship between mixed venous, central venous, and peripheral oxygen saturation in patients with advanced HF. These results have not been analyzed at the time of this publication. Secondary goals included investigation of correlations between biomarkers and PAC data. As an exploratory analysis, we also decided to evaluate outcomes, as defined below.

### Study population and design

Between December 2007 and August 2010, we prospectively enrolled 30 patients with advanced stage NYHA Class IV HF due to LV systolic dysfunction who were admitted to the Cardiac ICU at Massachusetts General Hospital for treatment guided by PAC monitoring. Patients were recruited for the study at the time of PAC placement.

Primary inclusion criteria were age ≥21 years and PAC insertion for management of HF by their attending cardiologist. At the time of PAC insertion all patients had evidence of decompensated HF manifesting in either: central venous pressure (CVP) >8 mmHg, pulmonary capillary wedge pressure (PCWP) >16 mmHg, systemic vascular resistance (SVR) >1200 dyn-sec/cm^5 ^or cardiac index <2 L/minute. Participants were excluded if they had known or suspected septic shock or bacteremia, active bleeding or hematocrit <24% at baseline, arterial oxygen saturation <90% at baseline despite supplemental oxygen therapy, femoral insertion of PAC, or if they were unable or unwilling to provide informed consent. At the time of study entry, detailed clinical data regarding past medical history, vital signs, medications, baseline laboratory data and left ventricular ejection fraction (EF) were collected.

Blood samples were collected twice daily, in the morning and evening, from the distal PAC port for the first 48 hours with corresponding hemodynamics and filling pressures noted at the time of the blood draws. All patients received standard HF management as recommended by contemporary guidelines [15]. Clinical management decisions about each patient, including timing regarding PAC removal and/or referral for LVAD placement or transplant, were made by the providing cardiologist who was blinded to biomarker concentrations, which were determined following completion of follow up.

### Biomarker analysis

Blood samples drawn for measurements of cardiac biomarkers were immediately spun and aliquots of plasma were stored at -80°C until analyzed. Testing for all of the assays was done in a random batch order, and testing personnel were unaware of the clinical status of the patients. Plasma concentrations of NT-proBNP, highly sensitive troponin I (hsTnI) and cys-C were determined on a Dimension Vista^® ^System, while plasma concentration of MPO was determined on a Dimension^® ^RxL Clinical Chemistry System from Siemens Healthcare Diagnostics (Tarrytown, NY, USA). In addition, a sensitive research prototype TnI (eTnI) assay was also run on the Dimension Vista^® ^System. The NT-proBNP, hsTnI and eTnI assays are all one-step sandwich chemiluminescent immunoassays based on LOCI (luminescent oxygen channeling assay) technology. The cys-C assay is a particle-enhanced immunonephelometric assay, and the MPO assay is a one-step sandwich enzyme immunoassay utilizing chrome particles. Plasma concentrations of sST2 were measured using the highly-sensitive Presage ST2^® ^Assay (Critical Diagnostics, San Diego, CA, USA) that has previously been validated [16]. Calibration of the assays was according to the manufacturer's protocol.

### Outcomes

Serum concentrations of the investigated biomarkers (NT-proBNP, hsTnI, eTnI, cys-C, MPO, and sST2) were hypothesized to predict outcomes in 30 heart failure patients admitted to an intensive care unit for PAC guided therapy. The primary outcome measure was failure to achieve individual goals for specific hemodynamic variables (CVP, PCWP, SVR) as part of PAC guided HF management within 48 hours after ICU admission, PAC removal or ICU discharge. Goals were pre-specified according to standard of care [17], and included: CVP <8 mm Hg, PCWP <16 mm Hg and SVR <1200 dyn-sec/cm^5^. Each hemodynamic variable (CVP, PCWP and SVR) was examined as a discrete element of PAC directed therapy.

In addition to response to PAC guided therapy, patients were clinically followed for events (defined as all-cause mortality, LVAD implantation, or heart transplantation) for 90 days, which was evaluated as a secondary outcome. Outcomes were ascertained from available medical records; mortality was confirmed via a Social Security Death Index database search.

### Statistical analysis

Demographics and clinical and laboratory variables were compared with Student's t-test (in states of normality) and Wilcoxon rank-sum testing (where non-normal). Biomarker concentrations, cardiac filling pressures and hemodynamics at matched time points were assessed for correlation using the Pearson method.

The highest biomarker value during the first 48 hours was used for each patient to explore outcomes. Patients were categorized as a function of response or non-response to therapy guided by invasive monitoring. The ability of biomarkers to predict non-response, defined as failure to achieve filling pressure goals as described above, was assessed by receiver operating characteristic (ROC) analysis with area under the curve (AUC) calculated. The optimum cut-point was identified as the point on the ROC curve that maximized both sensitivity and specificity. In an effort to understand better the association between sST2 values and risk for non-response, we performed stepwise logistic regression analyses, utilizing all the variables in Table 1 in a univariate screen with a retention variable of 0.10 for inclusion in the multivariate model. Separate models were performed for non-response in CVP, PCWP and SVR. From these, odds ratios (OR) and 95% confidence intervals (CI) were generated. Given the lack of a clearly defined sST2 value for each non-response category, the biomarker was entered as a log-transformed, continuous variable.

**Table 1:** Baseline clinical characteristics of the study population and clinical characteristics stratified by event (Death/LVAD implantation/transplant) at 90 days.

Characteristic	All Patients (Number = 30)	No Event (Number = 13)	Event (Number = 17)	*P* ^a^
Age	57 (50 - 65)	57 (45 - 63)	61 (53 - 65)	0.36
Male sex, number (%)	24 (77)	9 (69)	15 (88)	0.36
Ejection fraction, %	21 (17 - 30)	28 (19 - 35)	19 (17 - 25)	0.08
**History, number (%)**				
Ischemic cardiomyopathy	14 (47)	6 (46)	8 (47)	0.82
Prior myocardial infarction	8 (27)	5 (39)	3 (18)	0.24
Hypertension	4 (17)	3 (23)	2 (12)	0.63
Diabetes mellitus	11 (37)	5 (39)	6 (35)	1.00
Ventricular tachycardia	12 (40)	2 (15)	10 (59)	0.03
Resuscitated sudden cardiac death	5 (17)	1 (8)	4 (24)	0.36
Smoking (past or present)	16 (53)	8 (62)	8 (47)	0.57
Atrial fibrillation	11 (37)	3 (23)	8 (47)	0.26
Chronic kidney disease	9 (30)	3 (23)	6 (35)	0.69
Hypothyroidism	7(23)	2 (15)	5 (29)	0.43
**Medications on Presentation, number (%)**				
β-blocker	24 (80)	11 (85)	13 (77)	0.67
ACE Inhibitor	13 (43)	5 (39)	8 (47)	0.72
ARB	7 (23)	3 (23)	4 (24)	1.00
Aldosterone antagonist	21 (70)	8 (62)	13 (77)	0.44
Loop diuretics	28 (93)	11 (85)	17 (100)	0.18
Digoxin	9 (30)	2 (15)	7 (41)	0.23
**Medications During Study, number (%)**				
Diuretics	29 (97)	12 (92)	17 (100)	0.43
Afterload reducing agents	13 (43)	7 (54)	6 (35)	0.46
Inotropic agents	25 (83)	9 (69)	16 (94)	0.14
**Physical Examination**				
Heart rate, beats/min	78 (70 - 90)	84 (71 - 93)	74 (70 - 82)	0.17
Systolic blood pressure, mmHg	97 (90 - 104)	99 (91 - 104)	96 (85 - 104)	0.37
Body-mass index, kg/m^2^	25 (22 - 28)	26 (24 - 29)	24 (23 - 27)	0.25
**Laboratory Results**				
Blood urea nitrogen, mg/dL	25 (20 - 39)	30 (18 - 45)	24 (20-40)	0.90
Creatinine, mg/dL	1.3 (1.1 - 1.8)	1.2 (0.9 - 1.9)	1.4 (1.2 - 1.9)	0.32
Hemoglobin, g/L	12.0 (10.1 - 12.7)	12.3 (11.9 - 12.7)	11.3 (9.8 - 12.7)	0.18
Sodium, mmol/L	138 (133 - 140)	140 (138 - 141)	134 (132 - 138)	0.01
**Hemodynamic Indices**				
Central venous pressure, mmHg	10 (8 - 17)	10 (8 - 14)	9 (8 - 17)	0.73
Pulmonary artery systolic pressure, mmHg	52 (46 - 56)	55 (52 - 60)	48 (36 - 51)	0.01
Pulmonary artery diastolic pressure, mmHg	23 (18 - 28)	26 (24 - 31)	21 (16 - 22)	0.01
Pulmonary capillary wedge pressure, mmHg	24 (17 - 28)	25 (23 - 28)	23 (15 - 28)	0.31
Systemic vascular resistance, dyn-s/cm^5^	1,366 (993 - 1,590)	1,484 (974 - 1,666)	1,281 (997 - 1,557)	0.65
Cardiac index, L/min/m^2^	2.0 (1.7 - 2.4)	2.1 (1.7 - 2.4)	2.0 (1.8 - 2.3)	0.73
**Biomarker Results**				
sST2, ng/mL	148 (88 - 226)	87 (66 - 145)	183 (112 - 258)	0.02
NT-proBNP, pg/mL	5,205 (2,591 - 10,021)	4,437 (2,943 - 10,185)	5,388(2,287 - 13,840)	0.77
hsTnI, ng/mL	0.10 (0.04 - 0.20)	0.03 (0.02 - 0.05)	0.05 (0.02 - 0.17)	0.32
eTnI, ng/mL	49.9 (24.0 - 140.4)	29.6 (21.9 - 79.4)	60.6 (28.5 - 214.2)	0.20
cys-C, ng/mL	1.85 (1.11 - 2.16)	1.42 (0.95 - 2.23)	1.93 (1.50 - 2.63)	0.27
MPO, pM	860 (513 - 1353)	1,218 (588 - 1813)	803 (455 - 1138)	0.23
**Prognostic Indices**				
SAPS II	22 (18 - 29)	21 (18 - 29)	23 (18 - 29)	0.78

^a^P value for difference between those with an event and those without. Continuous variables are expressed as median and interquartile range. ACE, angiotensin converting enzyme; ARB, angiotensin II receptor blocker; BUN, blood urea nitrogen, cys-C, cystatin C; eTnI, experimental research prototype Troponin I; hsTnI, highly sensitive Troponin I; LVAD, left ventricular assist device; MPO, myeloperoxidase; NT-proBNP, amino terminal cleavage fragment of B-type natriuretic peptide; SAPS II, Simplified Acute Physiology Score II; sST2, soluble ST2.

In a similar fashion, biomarker concentrations were fitted to ROC curves for their ability to predict death, implantation of an LVAD, or heart transplantation within 90 days, and compared to the Simplified Acute Physiology Score (SAPS) II [18]. Then, all factors listed in Table 1 were tested for univariate prediction of events (death, LVAD implantation or heart transplantation), and significant covariates are reported. Only significant univariate predictors were entered into an age-adjusted Cox proportional hazards model. From the Cox model, hazard ratio (HR) and 95% CI were generated. The cumulative incidence of death, LVAD or heart transplantation was also estimated using the Kaplan-Meier method with curves compared with the log-rank test. If a patient experienced more than one event within the 90 days, for example, cardiac transplantation and death, only the first event was included in analysis. All tests were two-sided, and a *P*-value < 0.05 was considered statistically significant. All statistics were performed with SPSS (Chicago, IL, USA), STATA (College Station, TX, USA), or MATLAB (Natick, MA, USA) software.

## Results

### Baseline clinical and hemodynamic characteristics

A total of 149 patients were deemed potentially eligible for the study and 30 patients were enrolled; 119 of the eligible patients were excluded primarily for the following reasons: anemia, intubation, and refusal for participation.

Characteristics of the study population at presentation (*n *= 30) are detailed in Table 1. The median age was 57 (interquartile range (IQR) = (50 to 65)) years, the majority were men (77%), and the subjects had marked LV systolic dysfunction (median LVEF 21%). The majority of patients were considered candidates for heart transplantation. The majority of patients were on optimal HF medical therapy; when not on such therapy, the usual comorbid reasons for its cessation at this stage of disease were present, including the need for inotropic agents or severe renal insufficiency (mean serum creatinine 2.0 mg/dL). Clinical follow up for outcomes was determined in all 30 patients and 16 out of 30 patients had biomarker and hemodynamic data for each time point, with the majority of patients (77%) with data for three of the four time points. Patients had the PAC removed a mean of three days following ICU admission.

### Filling pressures, hemodynamics, and interventions

Baseline hemodynamic measurements revealed elevated cardiac filling pressures (CVP 10 (8 to 17) mmHg, and PCWP 24 (17 to 28) mmHg). Patients also had evidence of systemic vasoconstriction with elevated SVR 1,366 (993 to 1,590) dyn·s/cm^5^. The cardiac index was also decreased in the study population; 50% of the patients had a cardiac index less than 2.0 L/min/m^2^. At the discretion of the primary cardiologist who was blinded to biomarker measurements, nearly all the patients were treated with diuretics (97%), the majority was given inotropic agents (83%) to augment contractility and fewer than half were given afterload reduction (43%). Table 1 details the specific HF treatments applied to the study population.

### Biomarker concentrations and correlates

Within the first 48 hours following placement of the PAC, the median (IQR) sST2 concentration was 129.5 ng/mL (86.8 to 226.9), NT-proBNP was 5,205 (2,511 to 11,204) pg/mL, hsTnI was 0.036 (0.00 to 0.12) ng/mL, eTnI was 49.9 (22.4 to 157.8) pg/mL, cys-C was 1.85 (1.07 to 2.16) ng/mL and MPO was 860 (500 to 1,400) pM.

Significant positive correlations were observed between NT-proBNP and cys-C (r = 0.421; *P *= 0.023), and as would be expected, between hsTnI and eTnI (r = 0.967, *P *< 0.001). Modest correlations were observed for cys-C, MPO, and eTnI. Notably, no other significant correlations between markers were detected.

With respect to correlations between biomarkers and filling pressures, on Day 1 morning measurements, pulmonary artery systolic pressure negatively correlated with cys-C (r = -0.52, *P *= 0.039) and PCWP negatively correlated with eTnI (r = -0.55, *P *= 0.049). On Day 1 evening measurements, CVP correlated with MPO (r = 0.475, *P *= 0.019). There was a significant correlation observed between sST2 and CVP on Day 2 (r = 0.73, *P *< 0.001) as well as the highest sST2 in the first 48 hours and Day 1 morning CVP (r = 0.58, *P *= 0.009). A trend toward inverse correlation with sST2 and SVR was present (r = -0.436, *P *= 0.071). There were no significant correlations with sST2 and PCWP or cardiac index.

### Biomarkers and failure to respond to 'tailored therapy'

Following hemodynamic and filling pressure guided therapy, within 48 hours after ICU admission 12 patients (40%) failed to achieve the pre-specified CVP goal (CVP <8 mmHg) as per standard of care, The standard goal of PCWP <16 mmHg was not met by 18 patients (60%) and 14 patients (47%) failed to lower SVR below 1,200 dyn-s/cm^5^; such patients were thus also identified as non-responders.

Using ROC analysis of multiple biomarkers to predict non-response, we found that the maximal concentration of sST2 during the first 48 hours after enrollment was the best predictor for failure of improvement in filling pressures as shown in Table 2, while only NT-proBNP weakly predicted improvement in SVR and did not predict response to CVP or PCWP; other markers showed no association with therapy response. The optimal cut-point for sST2 concentration for the prediction of lowered CVP based on the ROC analysis was 171 ng/mL (sensitivity 83%, specificity 78%), which was superior to the median value, 129 ng/mL (83% sensitivity, 67% specificity) as shown in Table 3.

**Table 2:** Receiver operating characteristic analysis of all biomarkers for predicting response to pulmonary artery catheter guided therapy.

Biomarker	AUC	95% CI	*P*
**CVP non-response^a^**			
sST2	0.82	(0.66 - 0.98)	< 0.001
NT-proBNP	0.64	(0.42 - 0.85)	0.10
hsTnI	0.56	(0.34 - 0.77)	0.30
eTnI	0.51	(0.29 - 0.74)	0.45
cys-C	0.70	(0.51 - 0.90)	0.02
MPO	0.48	(0.24 - 0.72)	0.57
**PCWP non-response^b^**			
sST2	0.68	(0.45 - 0.91)	0.06
NT-proBNP	0.63	(0.40 - 0.86)	0.13
hsTnI	0.53	(0.31 - 0.74)	0.41
eTnI	0.52	(0.29 - 0.74)	0.44
cys-C	0.62	(0.37 - 0.87)	0.18
MPO	0.52	(0.31 - 0.74)	0.42
**SVR non-responsec**			
sST2	0.59	(0.38 - 0.80)	0.19
NT-proBNP	0.69	(0.48 - 0.89)	0.04
hsTnI	0.40	(0.19 - 0.61)	0.82
eTnI	0.39	(0.17 - 0.61)	0.84
cys-C	0.45	(0.23 - 0.67)	0.67
MPO	0.39	(0.18 - 0.60)	0.85

^a^AUC for CVP non-response was significantly higher for sST2 as compared to cTnI (*P *= 0.03), and eTnI (*P *= 0.02), no significant difference between AUC for sST2 and cys-C/NT-proBNP/MPO; ^b^No significant difference among AUCs for PCWP non-response; ^c^No significant difference among AUCs for SVR non-response. AUC, area under the curve; CI, confidence intervals; cys-C, cystatin C; eTnI, experimental research prototype Troponin I; hsTnI, highly sensitive Troponin I; MPO, myeloperoxidase; NT-proBNP, amino terminal cleavage fragment of B-type natriuretic peptide; sST2, soluble ST2.

**Table 3:** Operating characteristics of sST2 for predicting response to pulmonary artery catheter guided therapy

Outcome and cut-off point	Sensitivity	Specificity	PPV	NPV
**CVP non-response**				
129 ng/mL (median)	83%	67%	63%	86%
171 ng/mL (ROC optimal)	83%	78%	71%	88%
**PCWP non-response**				
129 ng/mL (median)	67%	67%	75%	57%
98 ng/mL (ROC optimal)	89%	58%	76%	78%
**SVR non-response**				
129 ng/mL (median)	57%	50%	50%	57%
105 ng/mL (ROC optimal)	71%	47%	53%	64%

CVP, central venous pressure; NPV, negative predictive value; PPV, positive predictive value; PCWP, pulmonary capillary wedge pressure; ROC, receiver operator characteristic; sST2, soluble ST2; SVR, systemic vascular resistance.

Considered dichotomously [See Additional file 1, Figure S1], median (IQR) concentrations of sST2 were significantly higher among CVP non-responders compared to responders (225.3 (173.8 to 277.1) versus 104.6 (65.2 to 154.3) ng/mL; *P *= 0.003). Patients whose PCWP failed to respond also trended toward higher median sST2 concentrations (181.7 (107.5 to 226.9) versus 88.2 (63.8 - 238.8) ng/mL; *P *= 0.05). There was no significant difference in median sST2 concentration between patients who met SVR goals and SVR non-responders (111.0 (78.2 to 217.6) versus 171.2 (94.9 to 263.7) ng/mL; *P *= 0.19).

In multivariate logistic regression analyses, sST2 values within the first 48 hours of ICU admission were a strong predictor of CVP non-response (OR = 52.4; 95% CI = 1.55 to 1,777.9, *P *= .03). The only other variable predictive of CVP non-response was prevalent atrial arrhythmia (OR = 41.9; 95% CI = 1.40 to 1,249.3, *P *= .03). sST2 values were not independent predictors of failed lowering of PCWP (OR = 2.72; *P *= .17) or improved SVR (OR = 2.43; *P *= .22).

### Risk of all-cause death, LVAD placement or cardiac transplantation

Over a follow-up period of 90 days, follow up was complete in 100% of patients. There were six deaths (20%), two LVAD placements (7%), and nine cardiac transplantations (30%); the combined outcome was reached in 17 patients (57%).

Table 1 compares the baseline characteristics and biomarker levels of patients who experienced an event and those who were event-free. The clinical attributes described in Table 1 include some of the variables that previously have been shown to be related to ICU mortality as part of the SAPS II prognostic score [18,19], such as age, blood pressure, heart rate, sodium and blood urea nitrogen. As seen in Table 1, there was no significant difference in SAPS II scores between those who had an event and those who remained event-free (21 (18 to 29) versus 23 (18 to 29); *P *= 0.78). While all biomarkers tended to show numerically higher concentrations in those patients who suffered adverse events, only sST2 concentrations were significantly higher (183.1 (112.8 to 258.4) versus 86.9 (66.0 to 145.6) ng/mL, *P *= 0.02; Table 1). Similarly, in ROC analyses only sST2 had an AUC significantly different from non-discrimination for the combined endpoint (Table 4; AUC = 0.76; 95% CI = 0.59 to 0.93; *P *= 0.001); this was superior to the SAPS II score (AUC = 0.53; 95% CI = 0.31 to 0.75; *P *= 0.40).

**Table 4:** Receiver operator characteristic curve analysis of all biomarkers for predicting events (death/LVAD implantation/transplant).

Biomarker	AUC	95% CI	*P*
sST2	0.76	(0.59 - 0.93)	0.001
NT-proBNP	0.53	(0.32 - 0.74)	0.38
hsTnI	0.61	(0.40 - 0.81)	0.15
eTnI	0.64	(0.44 - 0.84)	0.08
cys-C	0.63	(0.42 - 0.83)	0.11
MPO	0.63	(0.43 - 0.83)	0.10

AUC, area under the curve; CI, confidence intervals; cys-C, cystatin C; eTnI, experimental research prototype Troponin I; hsTnI, highly sensitive Troponin I; MPO, myeloperoxidase; NT-proBNP, amino terminal cleavage fragment of B-type natriuretic peptide; sST2, soluble ST2.

**Figure 1:**
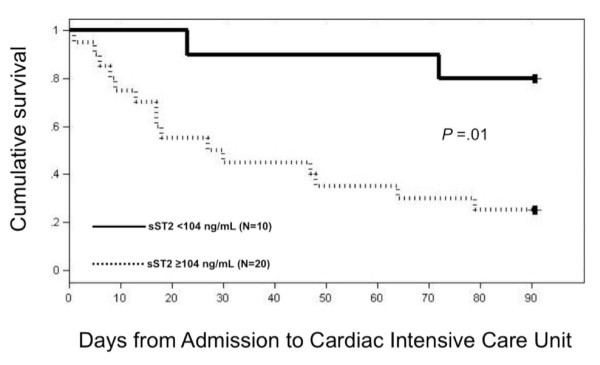
**Event free survival at 90 days according to sST levels**. Kaplan-Meier plot illustrates the incidence of death, LVAD, or heart transplantation among patients admitted to an intensive care unit for pulmonary artery catheter guided therapy according to sST2 levels. *P *= .01 by log rank test. LVAD, left ventricular assist device; sST2, soluble ST2.

As shown in Kaplan-Meier curves, the risk associated with an sST2 ≥104 ng/mL was present immediately from ICU admission and remained quite significant to the horizon of follow-up (Figure 1; log-rank *P *= 0.01).

In univariate analyses, sST2 was the only novel biomarker associated with a higher risk of experiencing an event. Adjusting further for age in a Cox proportional hazards model, the only variables that remained independent predictors of death, need for LVAD implantation or heart transplantation within the ensuing 90 days, were sodium <135 mmol/L (HR = 4.60; 95% CI 1.54 to 13.79; *P *= 0.006) and sST2 ≥104 ng/mL in the first 48 hours of ICU admission (HR = 5.53; 95% CI 1.20 to 25.42; *P *= 0.03). We thus examined the additive value of an elevated sST2 and hyponatremia for prognosis as shown in Additional file 2, Figure S2. At the end of the follow-up period, nearly all the patients with both hyponatremia and elevated sST2 had an event.

## Discussion

Although biomarkers are well-established for risk prediction in patients with typical HF presentations, their use in very advanced stages of the diagnosis, such as in hospitalized patients awaiting transplantation, is less explored. This is not without ramifications as, at such later stages of HF, information regarding prognosis can significantly influence decision-making such as more expedited LVAD placement or heart transplant. In this study, we examined the prognostic ability and correlation with hemodynamic indices of six biomarkers reflecting diverse pathophysiologic processes in a population of patients suffering from severe HF undergoing PAC-directed therapy. We hypothesized that biomarkers reflecting a broad range of pathophysiology in severe HF might provide incremental prognostic information in such patients, which might be theoretically leveraged for clinical decision making.

Consistent with prior studies, we found most biomarkers examined did not generally correlate with filling pressures [20]. An exception was sST2, which strongly correlated with CVP. While nearly all markers examined were less useful for predicting failure of 'tailored' therapy or events such as death or need for mechanical support or transplantation, concentrations of sST2 (held to reflect myocardial fibrosis and remodeling) within the first 48 hours following ICU admission appeared to predict both failure of PAC-based HF treatment as well as death or need for LVAD placement or transplantation.

The function of sST2 as a biomarker of mechanical stress was first demonstrated *in vitro *after up-regulation of its gene transcript was noted in conditions of myocardial strain [21] and has further evolved with the identification of its ligand, IL-33, which is also induced by myocardial strain. IL-33/ST2 signaling has been shown to be cardioprotective *in vivo*, with IL-33 acting as an anti-fibrotic, anti-apoptotic, and anti-hypertrophic paracrine hormone. IL-33/ST2 signal activation is thought to occur in response to cardiac strain, with sST2 as a 'decoy' receptor for IL-33; in the context of abnormal sST2 release, the protective effects of IL-33 are damped, leading to a risk for adverse cardiac remodeling and death [22-24]. sST2 has emerged as a powerful prognostic biomarker in various HF populations [9-11,25]; we now specifically extend the prognostic value of sST2 in an important population, namely those admitted to the ICU for advanced HF therapy. This is the first such specific study of sST2 in this context. Given the high risk and extensive cost-outlays associated with patients at this stage of disease, one could envision measurement of sST2 prior to the initiation of PAC guided therapy to aid a clinician's decision-making; a markedly elevated value might trigger more direct referral to mechanical support at a time prior to gross clinical instability.

Noteworthy of mention, our patient population demonstrated elevation of sST2 concentrations considerably higher than in previously reported populations [9-11,25], reflecting the unique nature of the study cohort. In addition, our subjects had considerable biochemical disarray as evidenced by the gross abnormalities of the other markers studied; this presumably diluted the ability of other biomarkers to recognize risk for failure of PAC guided therapy or 90 day complications. Interestingly, although not originally in the list of biomarkers evaluated, hyponatremia remained a predictor of adverse outcome even after adjustment, and when combined with sST2 identified patients at greatest risk for adverse outcomes. Hyponatremia has been well described as an independent predictor of mortality in HF [26,27], including in study populations of advanced HF similar to ours [28]. The rationale for expanding the analysis to consider hyponatremia with sST2 was based on the fact that HF patients with low sodium have more severe upregulation of the renin-angiotensin-aldosterone system (RAAS) [29,30], and angiotensin II (AngII) produced during RAAS activation has recently been studied for its role in mediating cardiac fibrosis. Acting in an autocrine and paracrine fashion, AngII activates signaling pathways including mitogen-activated protein kinases (MAPK), reactive oxygen species (ROS), and nuclear factor κB (NF-κB), all of which mediate cardiac hypertrophy, inflammation and fibrosis [31,32]. Sanada *et al. *demonstrated that IL-33 blocks NF-κB activation by AngII and co-treatment with sST2 reversed the inhibition of NF-κB, allowing unchecked development of hypertrophy [22]. Furthermore, IL-33 also suppresses both AngII-induced MAPK pathways and ROS generation [22]. The entwined relationship of ST2 signaling and AngII in cardiac hypertrophy/fibrosis coincides with our results demonstrating enhanced prognostic value of hyponatremia and elevated sST2 levels.

With each new prognostic biomarker developed, the relevant challenge from a clinical perspective is to elucidate whether the information provided expands on what is already known about the patient, and whether such data can be used to mitigate a patient's risk. Notably, similar with at least one other study in patients with severe HF [33], the SAPS II score did not provide substantial prognostic data, and was considerably inferior to sST2 for this purpose. Given the more widespread availability of biomarkers and relative ease of interpretation compared to more complex scoring systems, one could envision an approach of biomarker-guided or biomarker-supported therapy decision-making for advanced HF patients. Such decision-making has gained popularity in other disciplines but has yet to greatly gain momentum in cardiovascular medicine. Given its rising rates of morbidity and mortality as well as cost, HF is a promising cardiovascular diagnosis to explore this approach. A paucity of data exists for biomarkers specific to the most advanced stage HF patients (where treatment decision-making carries significant importance) but our results set the stage for a larger analysis similar in design.

This study has several important limitations. First, this is a small, single center study, which increases the risk of a Type II error; our hypothesis-generating study has set the stage for larger ICU based cohort studies, such as those that we are currently executing. Additionally, while the initial reason and powering for execution of this study was to evaluate the relationship among oxygen saturations drawn from the periphery, right atrium and pulmonary artery in patients with HF, we explored a secondary outcome measure (correlations to PAC data), while also examining cardiovascular event rates. The *post-hoc *decision to change the outcome measures is an additional limitation of this study, rendering our results more exploratory. Referral bias is another potential limitation of this study as all patients were enrolled after an attending cardiologist had determined them to require PAC-guided therapy. This may be reflected in our outcome rate, which was higher than in previously described populations of similar patients undergoing PAC guided therapy [3,17]. The highly specialized nature of our study population is worth noting. Finally, our study results may not be generalizable as our study population was predominantly male, which may not accurately reflect an ICU population.

## Conclusions

In summary, our data establishes heretofore undefined hemodynamic and outcome associations with sST2 concentrations in an understudied population and extends the current knowledge base regarding sST2 and cardiovascular disease. More studies in larger cohorts of such patients should seek to confirm these findings and advance the potential of future clinical management that is 'tailored' to the individual patient.

## Key messages

• Plasma concentrations of the interleukin receptor family member sST2 are loosely correlated with filling pressures and hemodynamics in patients with severe HF. The strongest correlations appear to be with right-heart filling pressures.

• An elevated concentration of sST2 in patients with Stage D, class IV HF undergoing PAC-guided therapy was associated with inability to achieve tailored filling pressure goals.

• Among patients admitted for hemodynamic tailoring, an elevated sST2 level predicted death, LVAD implantation, or heart transplantation at 90 days.

• Future studies investigating the role of sST2 in guiding therapy in Stage D, class IV patients are indicated.

## Abbreviations

ACE: angiotensin converting enzyme; AngII: angiotensin II; ARB: angiotensin II receptor blocker; AUC: area under the curve; BNP: B-type natriuretic peptide; BUN: blood urea nitrogen; CI: confidence interval; CVP: central venous pressure; cys-C: cystatin C; EF: ejection fraction; eTnI: experimental research prototype Troponin I; hsTnI: highly sensitive Troponin I; HF: heart failure; HR: hazard ratio; IQR: interquartile range; LVAD: left ventricular assist device; MAPK: mitogen-activated protein kinases; MPO: myeloperoxidase; NF-κB: nuclear factor κB; NT-proBNP: amino terminal cleavage fragment of B-type natriuretic peptide; NPV: negative predictive value; NYHA: New York Heart Association; OR: odds ratio; PAC: pulmonary artery catheter; PCWP: pulmonary capillary wedge pressure; PPV: positive predictive value; RAAS: renin-angiotensin-aldosterone system; ROC: receiver operating characteristic; ROS: reactive oxygen species; SAPS: simplified acute physiology score; sST2: soluble ST2; SVR: systemic vascular resistance.

## Competing interests

Dr. Januzzi has received grant support and consulting income from Roche Diagnostics, Siemens Diagnostics, Critical Diagnostics, and Thermo-Fisher Diagnostics. Dr. Wang has received assay or grant support from Diasorin, LabCorp, Siemens Diagnostics, Critical Diagnostics, Thermo-Fisher Diagnostics and honoraria from Quest Diagnostics, Diasorin, and Roche Diagnostics. Dr. Wang is named as a co-inventor on patents relating to the use of metabolite and neurohormonal biomarkers in cardiometabolic risk prediction. Dr. Gantzer was an employee of Siemens. The remaining authors declare that they have no competing interests.

## Authors' contributions

RVS, TJW and JLJ designed the study. JLZ and RVS were responsible for enrollment and data collection. JLZ, HKG and JLJ were responsible for data interpretation and analysis. MLG was responsible for biomarker analysis of the NT-proBNP, hsTnI, eTnI, cys-C and MPO. All authors were involved in the writing of the manuscript and approved the final version.

## Supplementary Material

Additional file 1 **Figure S1. Concentrations of sST2 and (A) CVP non-response, (B) PCWP non-response, and (C) SVR non-response**. The pdf file contains plots comparing the median sST2 and interquartile ranges for CVP responders and CVP non-responders in panel A, PCWP responders and PCWP non-responders in panel B, and SVR responders and SVR non-responders in panel C. Click here for file

Additional file 2 **Figure S2. Rates of events at 90 days as a function of hyponatremia and elevated sST2**. The pdf file contains a chart depicting the percentage of subjects who experienced an event (death, LVAD implantation, or heart transplantation) at 90 days as a function of hyponatremia and/or elevated sST2. Click here for file
