# Supplementary figures and images for: Measurement of multiple biomarkers in advanced stage heart failure patients treated with pulmonary artery catheter guided therapy
Source: Crit Care. 2012 Jul 25;16(4):R135. doi: 10.1186/cc11440 (PMC3580720; doi:10.1186/cc11440)

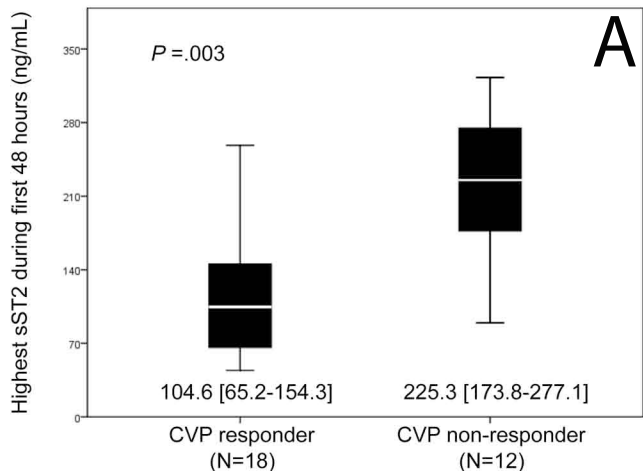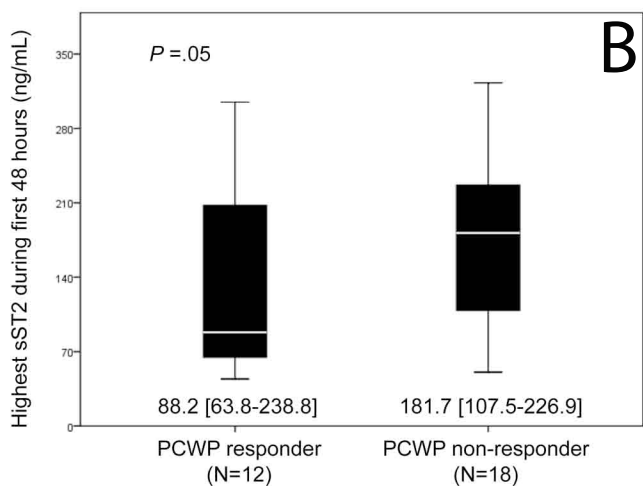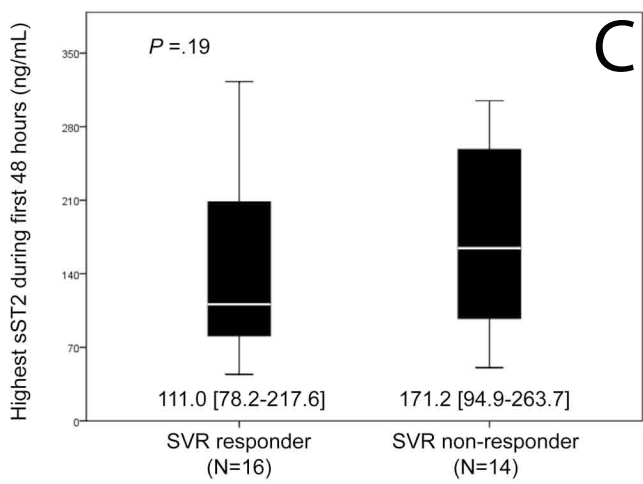

Supplement: Additional file 1 — Figure S1. Concentrations of sST2 and (A) CVP non-response, (B) PCWP non-response, and (C) SVR non-response. The pdf file contains plots comparing the median sST2 and interquartile ranges for CVP responders and CVP non-responders in panel A, PCWP responders and PCWP non-responders in panel B, and SVR responders and SVR non-responders in panel C. [file cc11440-S1.PDF]

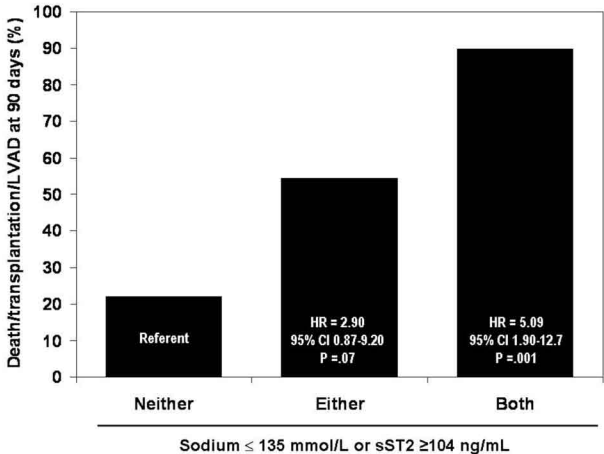

Supplement: Additional file 2 — Figure S2. Rates of events at 90 days as a function of hyponatremia and elevated sST2. The pdf file contains a chart depicting the percentage of subjects who experienced an event (death, LVAD implantation, or heart transplantation) at 90 days as a function of hyponatremia and/or elevated sST2. [file cc11440-S2.PDF]
